# The Antipsychotic D2AAK1 as a Memory Enhancer for Treatment of Mental and Neurodegenerative Diseases

**DOI:** 10.3390/ijms21228849

**Published:** 2020-11-23

**Authors:** Oliwia Koszła, Przemysław Sołek, Sylwia Woźniak, Ewa Kędzierska, Tomasz M. Wróbel, Magda Kondej, Aneta Archała, Piotr Stępnicki, Grażyna Biała, Dariusz Matosiuk, Agnieszka A. Kaczor

**Affiliations:** 1 Department of Synthesis and Chemical Technology of Pharmaceutical Substances with Computer Modeling Laboratory, Faculty of Pharmacy, Medical University of Lublin, 4A Chodzki St., 20-093 Lublin, Poland; sylwia.wozniak@umlub.pl (S.W.); tomasz.wrobel@umlub.pl (T.M.W.); magda.kondej@onet.pl (M.K.); piotrstepnicki93@gmail.com (P.S.); dariusz.matosiuk@umlub.pl (D.M.); 2 Department of Biotechnology, Institute of Biology and Biotechnology, University of Rzeszow, 1 Pigonia St., 35-310 Rzeszow, Poland; pp.solek@gmail.com; 3 Department of Pharmacology and Pharmacodynamics, Faculty of Pharmacy, Medical University of Lublin, 4A Chodzki St., 20-093 Lublin, Poland; ewa.kedzierska@umlub.pl (E.K.); grazyna.biala@umlub.pl (G.B.); 4 Department of Drug Design and Pharmacology, University of Copenhagen, Universitetsparken 2, DK-2100 Copenhagen, Denmark; 5 Department of Biopharmacy, Medical University of Lublin, 4A Chodzki St., 20-093 Lublin, Poland; aneta.archala94@gmail.com; 6 School of Pharmacy, University of Eastern Finland, Yliopistonranta 1, P.O. Box 1627, FI-70211 Kuopio, Finland

**Keywords:** hippocampus, neuron, central nervous system, neuroprotective activity, proliferation

## Abstract

The treatment of memory impairments associated with the central nervous system diseases remains an unmet medical need with social and economic implications. Here we show, that a multi-target ligand of aminergic G protein-coupled receptors with antipsychotic activity in vivo (D2AAK1) stimulates neuron growth and survival and promotes neuron integrity. We focused on the multilevel evaluation of the D2AAK1-related effects on neurons in terms of behavioral, cellular, molecular, and biochemical features in vivo and in vitro, such as memory-related responses, locomotor activity, tissue sections analysis, metabolic activity, proliferation level, neurons morphology, and proteins level involved in intracellular signaling pathways. *In silico* studies indicate that activation of calcium/calmodulin-dependent protein kinase I (CaMKI) may underline some of the observed activities of the compound. Furthermore, the compound increases hippocampal neuron proliferation via the activation of neurotrophic factors and cooperating signals responsible for cell growth and proliferation. D2AAK1 improves memory and learning processes in mice after both acute and chronic administration. D2AAK1 also causes an increase in the number of hippocampal pyramidal neurons after chronic administration. Because of its neuroprotective properties and pro-cognitive activity in behavioral studies D2AAK1 has the potential for the treatment of memory disturbances in neurodegenerative and mental diseases.

## 1. Introduction

Memory greatly contributes to our identity and individuality. This is why various forms of dementia, including the most common, Alzheimer’s disease (AD) [1], accounting for up to 75% of all dementia cases [2], are a frightening and strongly unwanted perspective for the senility. Moreover, memory disturbances often accompany other neurodegenerative diseases e.g., Parkinson’s disease and mental diseases, e.g., schizophrenia. It is, among others, associated with malfunction of the hippocampus, including protein aggregation, oxidative stress, or disturbances in the neurotransmitters levels [3]. Memory impairments associated with chronic central nervous system (CNS) disorders have social and economic implications. Indeed, neurological disorders are ranked as the leading cause of disability and comprise 16.8% of global deaths [4].

In general, the strategies to treat neurodegenerative diseases can be classified as neuroprotective or neuroregenerative. It can be expected that the treatment of neurodegenerative diseases, because of their complexity, require multi-target drugs [5]. Neuroprotection strategy is well-established and has been described for a number of small molecules with different molecular mechanisms: tacrine and its congeners [6], anxiolytic compound afobazol [7], and many natural compounds [8]. Interestingly, neuroprotective activity has been also reported for antipsychotic haloperidol, multifunctional D_2_-like dopamine, and sigma receptor subtype antagonist [9].

Handful of small molecule compounds have been reported to increase the number of neurons, their differentiation, or conversion of other cells into neurons [10,11,12,13,14,15]. Recently similar effects have been found for lithium chloride which is commonly used as a mood stabilizer [16].

As the hippocampus is necessary for the formation of new memories, it has been widely studied in AD and other dementias [17]. It has been demonstrated, that progressive degeneration of hippocampal neurons is responsible for short-term memory loss [18]. Moreover, microscopic changes in this brain part also precede the behavioral symptoms of AD [19]. Assessment of hippocampal neuron loss and hippocampus volume reduction have been proposed as the early diagnostic method of memory impairment in AD [20].

**Figure 1:**
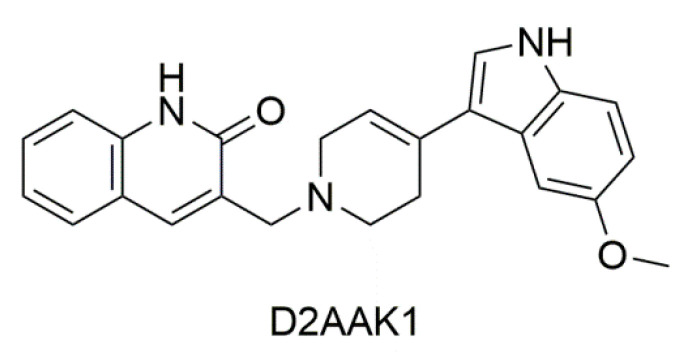
2D structure of D2AAK1.

In this work we focus on exploring new biological activities of 3-((4-(5-methoxy-1*H*-indol-3-yl)-3,6-dihydropyridin-1(2*H*)-yl)methyl)quinolin-2(1*H*)-one (D2AAK1) presented in Figure 1. This compound has been identified in structure-based virtual screening as a multi-target ligand of aminergic G protein-coupled receptors (GPCRs), namely dopamine D_1_, D_2_, and D_3_ receptors and serotonin 5-HT_1A_ and 5-HT_2A_ receptors [21]. It possesses antipsychotic, anxiolytic, and pro-cognitive activity in vivo [22] and has potential for the treatment of memory disturbances in mental and neurodegenerative diseases. In this work, we show that D2AAK1 stimulates neuron growth and survival and promotes neuron integrity most probably both through the activation of neurotrophic factors and cooperating signals responsible for cell growth and proliferation (including CaMKI kinase, mTOR kinase, Akt kinase, CREB protein and Erk1/2 kinase). Moreover, D2AAK1 improves memory and learning processes in mice both after acute and chronic administration. Finally, we demonstrate, that the compound increases the number of pyramidal layer neurons. During our investigation of D2AAK1, while evaluating its cytotoxicity, we observed enhanced proliferation of hippocampal neurons. This encouraged us to delve deeper into additional biological activity this compound might have. This led to the recognition of D2AAK1 as a prospective compound repurposed from an antipsychotic to a potential compound for the treatment of memory impairment in neurodegenerative and mental diseases.

## 2. Results

### 2.1. The Effect of D2AAK1 on Cell Metabolic Activity and Proliferation Level

**Figure 2:**
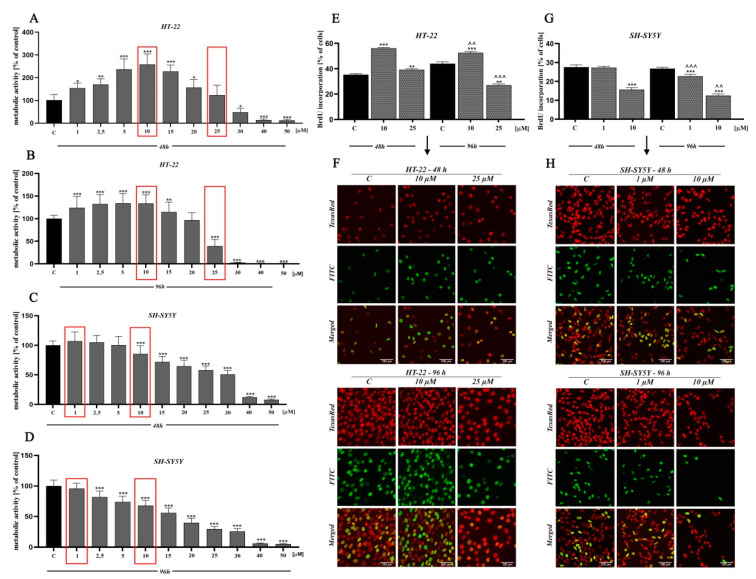
The effect of D2AAK1 on mouse hippocampal neurons (HT-22) and neuroblastoma cells (SH-SY5Y). Metabolic activity as measured with MTT assay with the selection of two concentrations for further investigation, marked in red frames (**A**–**D**) and BrdU assay (**E**–**H**) Red fluorescence-Texas Red, green fluorescence-FITC. Magnification of the objective lens 20×. Differences between control and test groups were assessed with one-way ANOVA and Dunnett a posteriori test. Bars indicate SD, *n* = 3, * *p* < 0.05; ** *p* < 0.01; *** *p* < 0.001 when compared between the control sample, not treated with the compound and the test sample incubated with the D2AAK1; ^^ *p* < 0.01; ^^^ *p* < 0.001 when compared between the incubation time (48 and 96 h).

In order to determine the potential cytotoxicity of D2AAK1 and its effect on the cell proliferation, we performed 3-[4,5-dimethylthiazole-2-yl]-2,5-diphenyltetrazolium bromide (MTT) and bromodeoxyuridine (BrdU) assay (Figure 2). In lower concentration range (1 µM–15 µM), we observed a significant increase of proliferation in mouse hippocampal neurons (HT-22 cell line) while neuroblastoma cells (SH-SY5Y cell line) proliferation at lower range were unaffected. As we expected, we also noted a dose-dependent decrease in cell proliferation and cell metabolic activity for higher concentrations range. Significant changes in cell activity were also associated with increased incubation time, as shown in Figure 2A–D. The observed drop-offs of cell viability correlated to a reduction in the viable cell number. Based on the MTT assay, we selected two compound concentrations for further research (marked in red frames). The first directly related to a high increase in cell metabolic activity (i.e., 10 µM-HT-22; 1 µM-SH-SY5Y) and the second associated with a slight decrease (toxic) (i.e., 25 µM-HT-22; 10 µM-SH-SY5Y).

Moreover, the reduction of cellular metabolic activity may be associated with impaired cell proliferation. Thus, we analyzed BrdU incorporation into newly synthesized DNA of actively proliferating cells. We observed an increase in HT-22 cells proliferation at low drug concentration, while the opposite effect was found at a higher concentration (Figure 2E,F). Interestingly, for SH-SY5Y cell line, we noted a reduction in cell proliferation compared to control for both high and low concentrations (Figure 2G,H).

### 2.2. D2AAK1 Influence on Neurons Morphology

**Figure 3:**
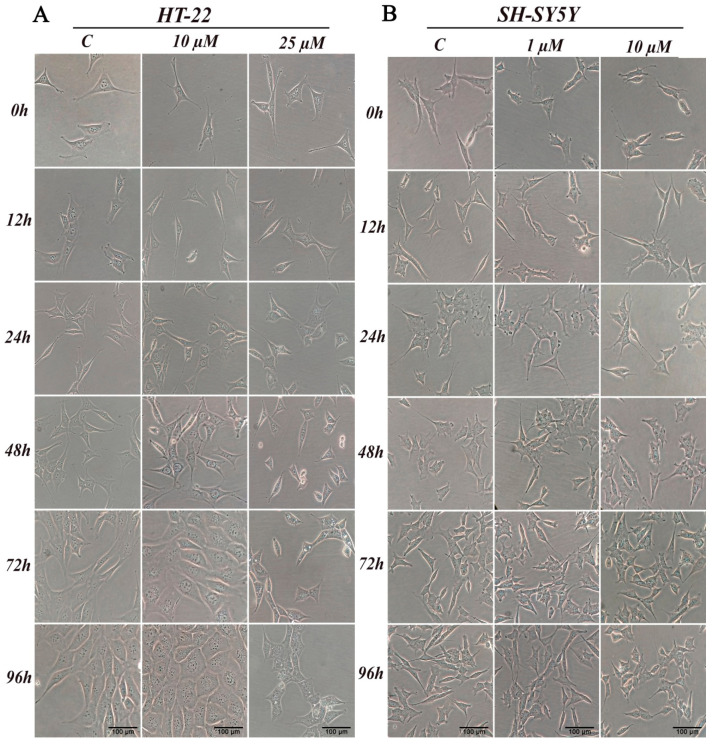
D2AAK1 mediated changes in cell morphology (**A**) HT-22 and (**B**) SH-SY5Y. Cells were treated for 96 h and digital images were captured. Magnification of the objective lens 10×.

Chemicals, in particular biologically active compounds, can interfere with normal cellular shape or size. In this study, we conducted an assessment of morphological changes over a 96-h D2AAK1 incubation. HT-22 cells were treated with 10 μM D2AAK1 and subsequently, their morphology was inspected (Figure 3A). The treated cells were characterized by increased size and more pronounced elongated shape compared to the control. Cells also exhibited longer dendrites and denser Nissl bodies. In turn, 25 μM D2AAK1 concentration resulted in shorter and more constricted cells, strong vacuolization as well as partial detachment from the substrate. Moreover, for this concentration, we observed that cell number decreased, which is directly related to their proliferation potential. All observed changes were more pronounced with the increasing incubation time. For SH-SY5Y cells, we did not observe any significant differences or morphological changes compared to the control (Figure 3B).

### 2.3. In Silico Probing of the Underlying Mechanism

In order to determine the possible molecular mechanisms responsible for the observed activities of D2AAK1, we used the molecular similarity approach as incorporated in PASS software [23] to compare D2AAK1 structure with compound structures of known bioactivities. We found, that CaMKI kinase delta that regulates axonal extension and growth cone motility in hippocampal and cerebellar nerve cells may be one of molecular targets of D2AAK1 (P_a_, probability that the compound is active: 0.520; P_i_, probability that the compound is inactive: 0.084). Although PASS software indicated D2AAK1 as an inhibitor of this kinase, this specific result can stem from a limited database of the software which may not contain activators of the enzyme. Therefore, it is imperative to experimentally verify if the predicted activity is indeed inhibitory and not activating, as recommended by the software developers [23].

### 2.4. Intracellular Signaling Pathways

**Figure 4:**
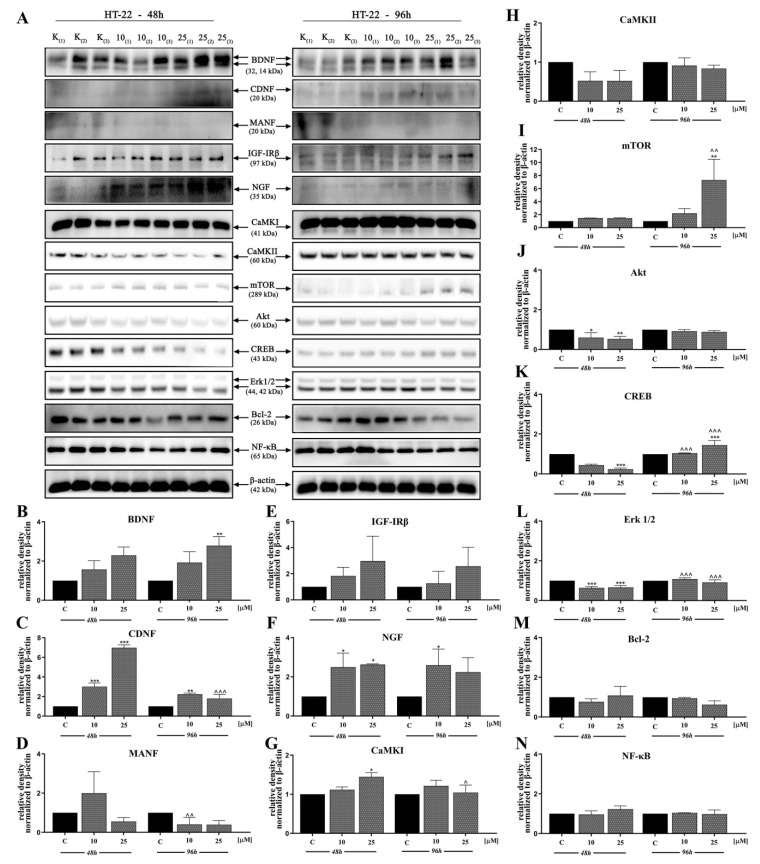
D2AAK1-mediated effect on HT-22 cellular protein pathways. Cells were treated for 48 and 96 h with two different concentrations of D2AAK1; representative immunoblots are shown (**A**). Western blot analysis of BDNF (**B**), CDNF (**C**), MANF (**D**), IGF- Irβ (**E**), NGF (**F**), CaMKI (**G**), CaMKII (**H**), mTOR (**I**), Akt (**J**), CREB (**K**), Erk 1/2 (**L**), Bcl-2 (**M**), NF-κB (**N**) synthesis level was performed. Bars indicate SD, *n* = 3, * *p* < 0.05; ** *p* < 0.01; *** *p* < 0.001 when compared between the control sample, not treated with the compound and the test sample incubated with the D2AAK1; ^ *p* < 0.05; ^^ *p* < 0.01; ^^^ *p* < 0.001 when compared between the incubation time (48 and 96 h).

**Figure 5:**
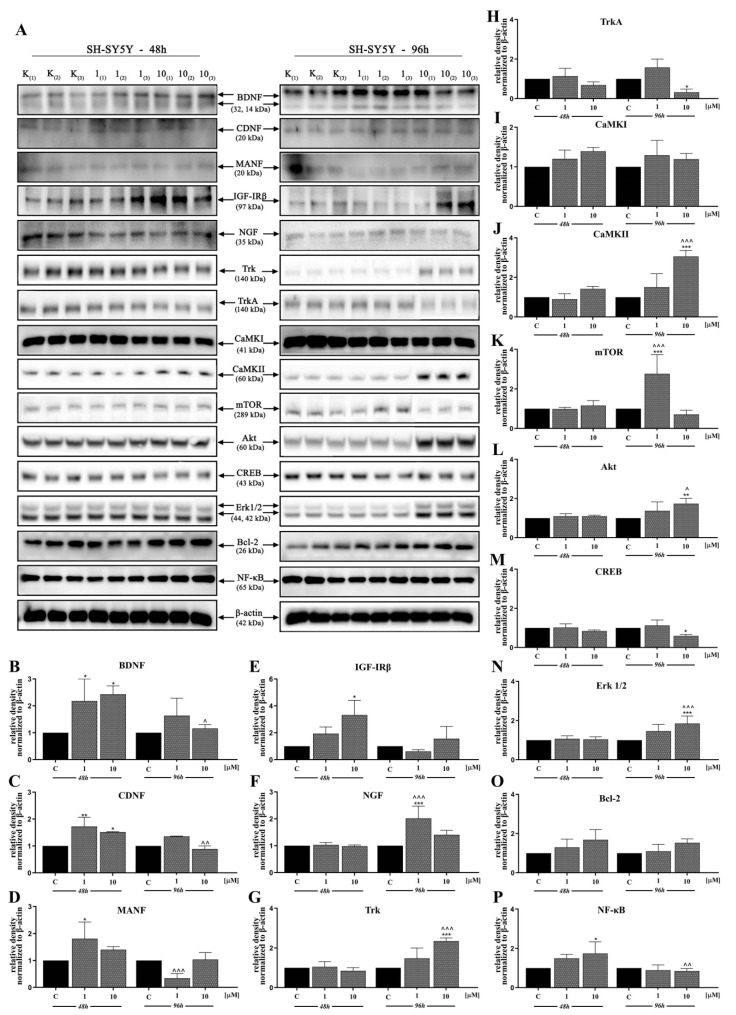
D2AAK1-mediated effect on SH-SY5Y cellular protein pathways. Cells were treated for 48 and 96 h with two different concentrations of D2AAK1. Representative immunoblots are shown (**A**). Western Blot analysis of BDNF (**B**), CDNF (**C**), MANF (**D**), IGF- Irβ (**E**), NGF (**F**), Trk (**G**), TrkA (**H**), CaMKI (**I**), CaMKII (**J**), mTOR (**K**), Akt (**L**), CREB (**M**), Erk 1/2 (**N**), Bcl-2 (**O**), NF-κB (**P**) synthesis level was performed. Bars indicate SD, *n* = 3, * *p* < 0.05; ** *p* < 0.01; *** *p* < 0.001 when compared between the control sample, not treated with the compound and the test sample incubated with the D2AAK1; ^ *p* < 0.05; ^^ *p* < 0.01; ^^^ *p* < 0.001 when compared between the incubation time (48 and 96 h).

In the next stage, we investigated the signaling pathways associated with the observed activities of D2AAK1 using Western blot analysis (Figure 4A–N and Figure 5A–P). D2AAK1 treatment promoted the pathway activation of neurotrophic factors BDNF (Figure 4B), CDNF (Figure 4C), MANF (Figure 4D), and NGF (Figure 4F). Consequently, the level of insulin-like growth factor receptor (IGF-IRβ) (Figure 4E), CaMKI (Figure 4G), and CaMKII (Figure 4H) were also elevated in all experimental sets. An up-regulation of Trk (Figure 5G) and TrkA (Figure 5H) neurotrophin receptor was observed, however, these results were found for SH-SY5Y cell line only.

Although the synthesis of proteins associated with plasticity and function of neuronal networks was disrupted in both cell lines treated with D2AAK1, the level of mTOR, Akt, CREB, and Erk 1/2 (Figure 5K–N) remained unaffected for SH-SY5Y line. However, we observed increased levels of these proteins for both cell lines after 96 h of incubation. The beneficial morphological changes accompanied by the activation of all described previous signaling pathways may also suggest the activation of cellular neuroprotective mechanisms. Western blot analysis against Bcl-2 and NF-κB (Figure 4M,N; Figure 5O,P) confirmed the slight up-regulation of both proteins, which enhances or even promotes proliferation and neuroprotection correlated with D2AAK1 treatment and even might prevent neuronal cell death.

### 2.5. The Effect of D2AAK1 on Learning and Memory Processes in Mice in Novel Object Recognition Test (NOR)

**Figure 6:**
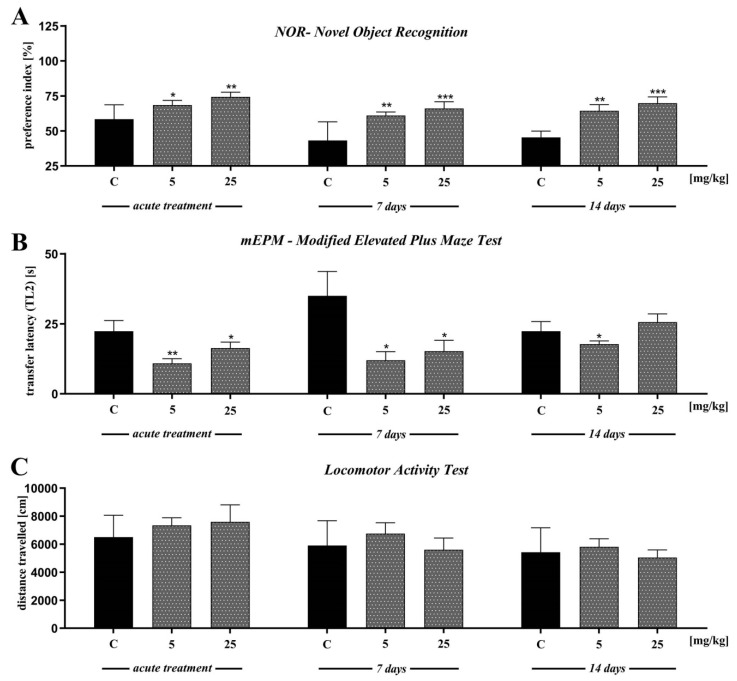
The effect of D2AAK1 on learning and memory processes (**A**,**B**) and locomotor activity (**C**). Mice were treated with two different doses of D2AAK1 for a different time period and NOR, mEPM, locomotor activity tests were performed. Bars indicate S.E.M, *n* = 8–10, * *p* < 0.05; ** *p* < 0.01; *** *p* < 0.001.

In the first trial (pre-test, day 2), we found no significant differences in preference index (PI) values between all groups (*p* > 0.05) (data not presented). Single-injection of D2AAK1 affects memory-related processes 24 h after drug administration [F(2,45) = 6.789; *p* < 0.01]. Accordingly, in the consolidation test trial we found that the acute administration of D2AAK1 significantly changed PI values at a dose of 5 mg/kg (*p* < 0.05) and at a dose of 25 mg/kg (*p* < 0.01) indicating an improvement of memory and learning processes. Similarly, the chronic administration (7 and 14 days) of D2AAK1 promoted pro-cognitive effects, as mice treated with this compound preferred novel object during test trial (T2) with respect to their counterparts on saline [F(2,27) = 8.940, *p* = 0.0010] and [F(2,21) = 11.89, *p* < 0.001], respectively. PI values increased (*p* < 0.01) after a dose of 5 mg/kg, and (*p* < 0.001) after a dose of 25 mg/kg (Figure 6A).

### 2.6. The Effect of D2AAK1 on Learning and Memory Processes in Mice Modified Elevated Plus Maze Test (mEPM Test)

In the first trial (pre-test), we found no significant differences in latency time 1 (TL1) values between all groups (*p* > 0.05) (data not presented). Single injection of D2AAK1 resulted in improvement of memory-related processes 24 h after drug administration in the mEPM test in mice [F(2,45) = 4.524; *p* < 0.01]. Indeed, we observed significantly changed latency time 2 (TL2) values compared to the saline-treated mice after both doses of D2AAK1 (*p* < 0.01 and *p* < 0.05, respectively) (Figure 6B).

In turn, we found no significant differences in TL1 correlated to chronic administration of D2AAK1 (7 and 14 days) (data not presented). Furthermore, 7 and 14 days chronic administration of D2AAK1 significantly changed TL2 values [F(2,21) = 4.650; *p* < 0.05] and [F(2,21) = 2.179; *p* = 0.1380], respectively. The chronic 7- days treatment induced pro-cognitive effects shortening the TL2 time period in the second trial (*p* < 0.05, for both doses used) while 14-days treatment induced pro-cognitive effects only after the lower dose of 5 mg/kg (*p* < 0.05) (Figure 6B).

In the last experiment, we evaluated the locomotor activity of mice exposed to acute or repeated D2AAK1 injection. We found no significant differences in the distance covered by the mice in all experiments (Figure 6C).

### 2.7. D2AAK1 Chronic Treatment-Induced Changes in Hippocampus

The chronic effect of D2AAK1 on hippocampal cells were determined using histological slides. Mouse brains were dissected, collected, and fixed as described in the Materials and Methods section. Histological examination of hematoxylin and eosin stained sections revealed normal, intact neurons with large nuclei and clear nucleoli. In addition, we observed unchanged thickness of pyramidal cell layers compared to the sections from control animals.

**Figure 7:**
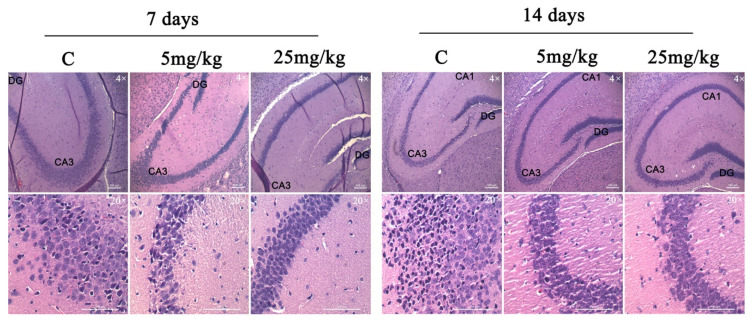
D2AAK1-mediated changes in hippocampus. The compound was administered for 7 and 14 days and histological slides were analyzed. Magnification of the objective lens 4× and 20×.

The analysis showed no changes related to the degradation of neurons, which include the lack of thickness reduction and loss of surface area of pyramidal cells. Moreover, no increase in the number of apoptotic cells or vacuolization of the cytoplasm was observed after the analysis of the image of the preparations. Importantly, we noticed the increase in the number of pyramidal neurons in sections from the D2AAK1-treated animals compared to the control animals. More specifically, the highest increase was found after 14 days of treatment with 25 mg/kg of D2AAK1 and this increase was observed in all the hippocampus regions studied (Figure 7.)

## 3. Discussion

The unique properties of D2AAK1, identified in structure-based virtual screening, result from the fact that it is in vivo active multi-target ligand of aminergic GPCRs and displays favorable effects on hippocampal cells.

Earlier animal studies [22] have shown, that D2AAK1 exerts antipsychotic and anxiolytic activity and is characterized by pro-cognitive effects. However, the exact molecular mechanisms underlying the effect of D2AAK1 on memory and learning processes remained elusive. Here, encouraged by the observed in vitro neuron growth, enhanced neuron survival and improved neuron integrity caused by D2AAK1, we provide for the first time novel mechanistic data of D2AAK1 action on learning and memory processes.

The dose-response effect is central to brain function understanding and CNS illness treatment. For this reason, our interest focused on low-dose stimulation (hormesis) and medium-dose inhibition (toxic threshold) using D2AAK1. In this regard, we observed an increase in metabolic activity of D2AAK1-treated HT-22 cells, in contrast to SH-SY5Y cells which were unaffected. The obtained results suggest, that the proliferative effect of D2AAK1 on the studied cell lines is highly selective. Selected cell lines (HT-22 and SH-SY5Y) exhibit neuronal like characteristics and serves as an experimental in vitro models for neurodegenerative diseases research. Moreover, only hippocampal neuronal cell proliferation increase was confirmed by BrdU proliferation assay. The fact that antipsychotics promote cell division was reported earlier [24,25]. It has also been proved that antipsychotic drugs improve the survival of newly generated nerve cells [26]. Therefore, increased proliferation of neurons may be beneficial for generating new neural junctions replacing lost or damaged cells and thus maintaining their proper function.

Furthermore, we found a number of beneficial cell morphological changes caused by D2AAK1, such as a more elongated shape and longer dendrites. In turn, all adverse changes in cell morphology occurred at high D2AAK1 concentrations, suggesting the activation of adaptive mechanisms related to cell survival under stress conditions [27]. Interestingly, there were no characteristic changes in cancer cell line. Thus, the obtained result suggests that D2AAK1 is selective toward healthy hippocampal cells vs. neuroblastoma cells. There are also reports that indicate that different antipsychotic medications may interact with neuroplasticity and morphological or cellular alterations [28,29,30].

Then, to study and explain the mechanistic pathways involved in the regulation of cell activity, proliferation, and neuron protection. PASS software indicated that CaMKI kinase that regulates axonal extension and growth cone motility in hippocampal and cerebellar nerve cells can be involved in the activity of D2AAK1. We confirmed this hypothesis using Western blot analysis. Moreover, we found D2AAK1 induced the upregulation of CaMKII in SH-SY5Y, while the increase of CaMKI level was observed in HT-22. Our findings underscore a central role of CaMK kinases family in signal transduction, protein synthesis, synaptic plasticity, neuronal development and behavior [31], suggesting that CaMK activity may be related to neurodegenerative disorders. These characteristics are in agreement with other observations where the synaptic plasticity is a key element for memory formation in the hippocampus [32]. Next, we noted that D2AAK1 induces CREB phosphorylation associated with long-term memory. Notably, this effect was observed only in the case of HT-22 cells and is in line with our previous results. Other authors clearly indicated the important role of the BDNF/CaMK/CREB signaling pathway in neuronal activation, proper synaptic plasticity, and further neurological disorders [33].

BDNF and other neurotrophins protect neurons against endoplasmic reticulum stress-induced cell death and also induce neurogenesis, synaptic plasticity during homeostatic adjustment and are involved in dopaminergic neurons protection [34]. Our previous studies demonstrated that D2AAK1 is a dopamine D_2_ receptor antagonist [22]. We used Western blot analysis to find D2AAK1 induced neurotrophic factors (BDNF, CDNF, MANF, NGF) up-regulation, which is also in agreement with earlier studies of the effect of antipsychotics on neurotrophic factors [35]. A high level of neurotrophins has been linked to neuron functions, cell survival and development, and capability of cells to repair. Additionally, neurotrophins regulate tropomyosin-related kinase (Trk) signaling pathways. We observed the upregulation of Trk and TrkA receptors, unexpectedly for SH-SY5Y cells only. The explanation for this result may be selective inhibition of receptor kinase signaling by D2AAK1 [36].

Additionally, we confirmed the activation of Erk1/2 and Akt/mTOR signaling pathways by D2AAK1. It was reported that BDNF has an ability to dephosphorylate Akt [37], which was confirmed in this study. Previous reports and our experiments demonstrated that inactivated Akt may also be dependent on mTOR and Erk 1/2. This possibility is in agreement with previous data [38]. Furthermore, impaired Akt-dependent signaling pathway affects neuronal connectivity and neuromodulation and can be associated with several disease states. Moreover, reduced but not obliterated activation of the PI3K/Akt/mTOR pathway has a key role in synaptic plasticity, neurotransmission, or stress responses including DNA repair [39].

Subsequently, we extended our research toward a molecular approach to signaling pathways important for the promotion of cell proliferation. We found that D2AAK1 treatment resulted in the up-regulation of IGF-IRβ and NF-κB but the level of protein synthesis was cell-specific. The low level of NF-κB transcription factor may indicate that during enhanced cell proliferation this protein is highly required but also suggests no D2AAK1 toxicity to hippocampus cells. In addition, longer incubation can lead to the accumulation of certain proteins. Data demonstrate that the expression of IGF-IRβ/NF-κB induces, supports, or targets Bcl-2 [40,41]. Additionally, it is well-known that Bcl-2 plays important neuroprotective functions [42], and NF-kB is a protein complex that can also participate in cell responses such as stress and free radicals [43]. Interestingly, we did not observe significant changes for these proteins. Thus, we hypothesize that the compound does not lead to cell damage. The most probable explanation of our results is the positive action of cell repair and adaptation mechanisms in the case of medium concentration of D2AAK1 and its neuroprotective effect.

In the next step of our research, we verified whether changes in the hippocampal cells were reflected in behavioral tests. We examined the effects of D2AAK1 on memory and learning processes in mice after acute and chronic administrations. In the present study, we used two different models of memory—mEPM and NOR paradigms.

Results obtained from the mEPM indicate that the acute, as well as chronic administration of D2AAK1, improves memory-related processes. Accordingly, an acute injection of D2AAK1 (5 and 25 mg/kg), applied immediately after the pretest may significantly and dose-dependently decrease TL2 values in the mEPM during the test trial, as compared to the control group. A similar effect was observed after chronic (7 and 14 days) treatment with D2AAK1 at a dose of 5 mg/kg while the dose of 25 mg/kg was effective only 7 days after treatment.

These pro-cognitive effects were also confirmed in the NOR test (often used to screen for memory enhancing or memory-disrupting compounds, including psychotomimetics and antipsychotics) both after acute and chronic treatment with D2AAK1 (5 and 25 mg/kg). Mice treated with the compound preferred N object during the test with respect to their counterparts on saline (control), so PI values in the test sessions were higher. The results from the chronic treatment indicate that, in this test used, D2AAK1 enhances memory consolidation after both doses, with comparable outcomes as that for the acute treatment.

This memory-improving effects of D2AAK1 in drug-naïve mice in the mEPM and NOR test are encouraging and may provide an additional benefit against cognitive dysfunction associated with schizophrenia since the compound elicits antipsychotic activity. Those results are even more significant, considering that most of the antipsychotic drugs in both preclinical and clinical cognitive studies elicit varying effects on normal cognitive function, i.e., having marked efficacy on cognition, like atypical ones (e.g., clozapine and olanzapine) or producing no effect or disturbing cognitive functions like typical antipsychotics (e.g., haloperidol) [44,45,46,47]. Thus, the new standards of treatment do not recommend classical neuroleptics but rather atypical drugs in schizophrenia, especially since they have a more favorable profile in the context of cognitive functions, attention, memory, verbal fluency [48,49,50]. However, taking into account that the effectiveness of antipsychotic drugs, aimed both at improving cognitive functions and schizophrenia-related disturbances is at best limited, it is urgently needed to elaborate a drug able to cure both symptoms, as these are still considered important unmet clinical needs.

Previous behavioral studies have demonstrated that among the brain structures, the frontal cortex and hippocampus are involved in visual learning and memory in the NOR [51,52,53]. This study was the first to demonstrate the enhancement of cognition by D2AAK1 as well as the stimulating effects of D2AAK1 on the proliferation of hippocampal neurons pointing to the unique properties of the compound. It is believed that an increase in neurogenesis in the adult hippocampus improves cognitive processes. Therefore, there are still attempts in seeking drugs influencing the growth of neurons, especially in the dentate gyrus, that would effectively improve memory [54,55].

To rule out the possibility of sedative effects of the D2AAK1 on animal behavior in mEPM and NOR tests, we performed the locomotor activity test. D2AAK1 at doses 5 and 25 mg/kg, did not have any influence on the locomotor activity of mice on any of the tested days (day 1, day 8, day 15). Thus, the presented pro-cognitive effects induced by D2AAK1 in both tests were not disturbed by changes in motility. Our findings from both behavioral tests are consistent with our other data and can suggest that the influence of D2AAK1 on CaMK kinases and neuronal development together with increased hippocampal neuron proliferation, may be consider one of the key mechanisms of improving memory and learning processes. Because of its neuroprotective properties and pro-cognitive activity confirmed in behavioral studies, D2AAK1, after acute and chronic administration, can be used for the treatment of memory impairment in neurodegenerative and mental diseases.

Collected mice brains, after behavioral studies, served to assess the changes in the hippocampus resulting from chronic administration of D2AAK1. We observed normal pyramidal neurons in all layouts studied, without any typical changes associated with degrading neurons. In addition, we observed an increase in pyramidal layer neurons because of D2AAK1 administration to mice. According to our assumptions, we noted a significant improvement in memory in mice after administration of the compound for 14 days at a dose of 25 mg/kg which reflected the results obtained from histological preparations where the largest increase in the number of neurons was also observed with the same parameters. Our findings further confirm that because of the important role of the hippocampus in the proper functioning of memory [56], D2AAK1 may be considered as a promising compound for the treatment of AD and memory disturbances, also related to schizophrenia.

## 4. Materials and Methods

### 4.1. Antibodies

Antibodies used were: BDNF (Cat# sc-546, RRID:AB_630940), CDNF (Cat# sc-162667, RRID:AB_10986132), MANF (Cat# sc-34562, RRID:AB_2227485), IGF-IRβ (Cat# sc-9038, RRID:AB_671793), NGF(Cat# MA5-32067, RRID:AB_2809361), Trk (Cat# 4609, RRID:AB_2800196), TrkA (Cat# 2508, RRID:AB_2797602), CaMKI (Cat# ab68234, RRID: AB_1140889), CaMKII (Cat# 3362, RRID:AB_2067938), mTOR (Cat# 2972, RRID: AB_330978), Akt (Cat# 9272, RRID:AB_329827), CREB (Cat# 701120, RRID: AB_2532397), p44/42 MAPK (Erk1/2) (Cat. #4695, RRID:AB_390779), Bcl-2 (Cat# sc-7382, RRID:AB_626736), NF- κB (Cat# PA5-37658, RRID:AB_2554266).

### 4.2. D2AAK1

D2AAK1 was obtained in-house according to a newly elaborated procedure (see Supplementary Information for synthesis details and spectral characterization). For in vitro studies, D2AAK1 was dissolved in DMSO to a 10 mM stock solution. Dilutions were prepared in full culture medium just before each assay from the stock solution. Cells were treated for 48 h and 96 h. The final DMSO concentration for in vitro studies was 0.5%. The percentage of DMSO was adjusted for each sample tested. We also compared the samples with w/o DMSO and was well tolerated with no observable toxic effects to both cell lines tested. For in vivo studies, D2AAK1 was also dissolved in DMSO to a final concentration of 0.1% and then diluted with a 0.5% aqueous solution of methylcellulose (tylose) and injected intraperitoneally (i.p.) at a volume of 10 mL/kg. The low concentration of DMSO has no toxic effect on the results of in vivo tests. Fresh D2AAK1 solutions were prepared on each day of experimentation. Control groups received vehicle injections at the same volume via i.p. route of administration at the respective time before the tests.

### 4.3. Cell Culture

The mouse hippocampal neuron line (HT-22) was purchased from Merck, CA (Cat# SCC129, RRID:CVCL_0321) and routinely maintained in the medium recommended by the manufacturer (DMEM, Corning, New York, NY, USA) with L-glutamine, 4.5 g/L glucose and sodium pyruvate. The neuroblastoma line (SH-SY5Y) was purchased from ATCC, NY (Cat# CRL-2266, RRID: CVCL_0019) and maintained in DMEM/F-12 (50/50, *v/v*%) with L-glutamine, 4.5g/L glucose and sodium pyruvate. Culture media were supplemented with 10% fetal bovine serum-FBS (Gibco by Life Technologies, Carlsbad, CA, USA) and antibiotic mix solution (100 U/mL penicillin, 0.1 mg/mL streptomycin). Cell lines were cultured in a humidified CO_2_ incubator (New Brunswick Galaxy 170R, Thermo Fisher Scientific, Waltham, MA, USA) at 37 °C in a gas mix containing 5% CO_2_ to maintain physiological pH. For each assay, cells were seeded at a constant density of 3.0 × 10^3^ cells/cm^2^ for the HT-22 and 3.0 × 10^4^ cells/cm^2^ for the SH-SY5Y.

### 4.4. MTT Assay

MTT assay was performed as described elsewhere [42]. Cells were seeded on a 96-well plate at standard density (see above). After ~24 h, cells were treated with various concentrations of D2AAK1 for 48 h and 96 h. After treatment, MTT solution was added at a final concentration of 5 mg/mL. After 4 h incubation at 37 °C, absorbance was measured at 590 nm (and 620 nm as a reference) using BioTek -Synergy ™ H1 microplate reader. The assay was performed in a concentration range of 1–50 µM

### 4.5. BrdU Assay

Cells were prepared in 96-well plates. After 48 h and 96 h incubation with D2AAK1 in concentration 10 μM or 25 μM for HT-22 and 1 μM or 10 μM for SH-SY5Y, BrdU reagent was added at a final concentration of 10 µM and left for 2.5 h. The cells were subsequently fixed with 3.7% formaldehyde for 15 min and washed with PBS buffer. Then 100 μL PBST was added as a permeabilization step. The contents of the wells were removed and 50 μL of 2 M HCl was added (denaturation stage), blocked with 1 % BSA in PBST, and incubated at 4 °C overnight with primary antibody anti-BrdU. The next day, propidium iodide (2 μg/mL) was added and incubated for 15 min at RT. Images were taken using an InCell Analyzer 2000.

### 4.6. Morphology Analysis

Morphological changes were assessed for both cell lines (in concentration 10 μM or 25 μM for HT-22 and 1 μM or 10 μM for SH-SY5Y) treated with D2AAK1. Images were taken after 12 h and every 24 h for 96 h using JuLI™ Br Live Cell Analyzer equipped with CCD camera. Morphological analysis was performed on the basis of the obtained images.

### 4.7. Activity Mechanism Prediction

The mechanism of biological activity of D2AAK1 was predicted using PASS [23] and Pharma Expert software.

### 4.8. Western Blot

Total protein extracts were prepared as described elsewhere [42] and Western blot was performed. Proteins were separated based on molecular weight using electrophoresis SDS-PAGE. After electrophoresis, the separated proteins were dry transferred using the iBlot 2 Transfer Stacks which contains the required buffers and transfer membrane (PVDF). The proteins were then blocked in milk diluted in TBST for 1 h to prevent nonspecific binding of the antibody. The proteins were then incubated with primary and secondary antibodies conjugated to HRP. Detection and localization of target proteins were performed using ECL Western blotting substrate Westar Supernova (Cyanagen, Bologna, Italy) and imaging system Azure Imager c400-60. The results were normalized to β-actin (GelQuantNET software, San Francisco, USA).

### 4.9. Animals

The experiments were carried out on naive male Swiss mice weighing 20–25 g, from a licensed breeder of the Center for Experimental Medicine in Lublin, Poland. Four animals were kept in a cage, under strictly controlled laboratory conditions (temperature maintained at 22  ±  1 °C, relative humidity at 50–60%) with an artificial 12/12 h light/dark regime (light on at 8:00 a.m.). Standard laboratory food (LSM, Agropol, Motycz, Poland) and filtered water were continuously available. The animals were randomly assigned to the experimental groups, containing 8–10 individuals. All experiments were conducted between 8:00 a.m. and 3:00 p.m., in accordance with the European Union Directive of 22 September 2010 (2010/63/EU) and Polish legislation acts concerning animal experimentation. License number 110/2019 was issued by the Local Ethics Committee for Animal Experiments, University of Life Sciences in Lublin, Poland on December 20^th^ 2019

### 4.10. Memory-Related Responses

**Figure 8:**
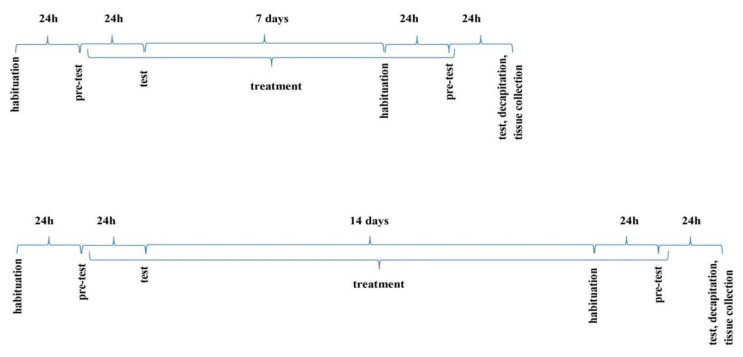
The schedule of administration of the compound D2AAK1 (i.p.) in the NOR and mEPM paradigms.

The NOR test was performed accordingly to the respective literature [57,58]. The apparatus was a plexiglass open box with the following dimensions: 63 cm long × 44.5 cm high × 44 cm wide. The box was illuminated with an intensity of 10 lx. Two objects of different shapes (block and ball) and material (wood or plastic), too heavy to be moved, were placed in a box. The habituation carried out the day before the test consisted in placing the animal in an empty box for 15 min to get used to the environment. On the next day (24h later–see the diagram–Figure 8), the animals were participating in pre-test: this trial (T1) lasted 5 min. The second trial (test trial, T2) was also 5-min long and was carried out after 24 h. On T1, mice were returned to the box with two identical objects (blocks made of wood). On T2, one of two familiar objects was replaced by a novel one (N), with different shape and material (plastic ball). If the mouse brought the nose a minimum of 2 cm to the object or touched it, this was considered exploratory behavior. The time of interest of each object during test sessions T1 and T2 was measured with a stopwatch by a blind observer.

Preference index (PI) was measured by comparing a time period, spent for exploration of the N object with time spent for exploration of the F object. Memory was evaluated with this index, calculated for each animal by the following formula: (N × 100)/(N + F), corresponding to the difference between exploration time periods for N and F objects, adjusted for the total exploration time period of both objects in T2. A higher PI value is considered to reflect pro-cognitive effects [59,60].

### 4.11. Modified Elevated Plus Maze Test (mEPM)

The mEPM test is conducted in a plexiglass apparatus, with crossed arms, two open (50 × 10 cm) and two enclosed (50 × 10 × 40 cm), elevated 50 cm above the floor. There is a 10 × 10 cm central platform in the middle [58,61]. The apparatus in the darkened room is only illuminated above the central platform by dim light of 10 lx. On day 1 (pre-test), the animal was placed on the end of the one open arm facing the central platform and the time of transfer to the closed arm was recorded (transfer latency 1, TL 1). If the mouse did not enter any enclosed arm within 90 s, it was placed there to freely explore the maze for another 60 s. Entering one of the arms was considered to be crossing the imaginary line separating this arm from the central platform. 24 h after the pretest, another experiment was carried out in which TL2 was measured. If the mice did not enter any of the enclosed arms within 90 s during this trial, TL 2 was recorded as 90 s. TL2 serves as an indicator of learning and memory processes. When a drug has an amnestic effect, it prolongs this value, and when it shortens it—it improves memory [61,62]. To avoid artifacts, the apparatus was cleaned after each mouse.

To observe acute effects of D2AAK1 on memory consolidation processes, the drug was administered immediately after TL1 trial (the 1st day) and 24 h later, the TL2 trial was performed (the 2nd day). To observe the chronic effects of D2AAK1 on memory consolidation processes, the drug was administered for 7 and 14 days, starting immediately after TL2 trial, and again, on the 8th and 15th day, the TL1 and 24 h later, the TL2 trial was performed.

During the acute treatment, the animals were allocated to the following drug treatment groups: D2AAK1 [5 and 25 mg/kg; by intraperitoneal (i.p.) injection] (*n* = 8–10), or vehicle (i.p.) (*n* = 8–10). The agents were administered at a volume of 10 mL/kg. Fresh drug solutions were prepared on each day of the experiment. Control groups received vehicle injections at the same volume and via the same route of administration.

For chronic treatment, animals were injected with D2AAK1 (5 and 25 mg/kg; or vehicle, once daily for 7 and 14 consecutive days). On the last day of treatment, D2AAK1 or vehicle was injected immediately after the pre-test and the rodents were retested 24 h later. The doses of D2AAK1 were chosen based on previously published results and our preliminary studies [22].

### 4.12. Locomotor Activity Test

The locomotor activity of individual mice was recorded using a device equipped with infrared emitters and detectors monitoring the movements of the animals (Opto-Varimex-4 Auto-Track, Columbus Instruments, Columbus, OH USA). To evaluate the locomotor activity of mice after acute administration, the compound D2AAK1 (5 and 25 mg/kg; i.p.) or vehicle (as a control) was administered 60 min before the test, and to evaluate the influence of tested compound D2AAK1 on spontaneous activity of mice after chronic treatment, D2AAK1 was administered to mice for 7 and 14 consecutive days. The motility of animals was measured on days 8 and 15 as the distance traveled (in cm), during 30 min. The apparatus was cleaned after each mouse with 10% ethanol.

### 4.13. Histological Examination of Hematoxylin and Eosin Stained Hippocampal Sections

Following the memory evaluation tests conducted after repeated administration (i.e., 7 and 14 consecutive days) of D2AAK1 (5 mg/kg; 25 mg/kg), mice were decapitated, and the whole brain from each animal was carefully taken out and rinsed with isotonic saline solution to remove blood. The collected brains of mice were fixed according to the procedure presented in Table S1 (Supplementary Information). The prepared tissues were embedded in paraplast blocks, then cut with a microtome (Leica Wetzlar, USA) into 5 µm thick scraps sections and applied to slides previously coated with APS.

In the next step, tissue sections were hydrated (to introduce water into the tissue section) by passing the slides slowly through a series of decreasing concentrations of alcohol (see Table S2 in Supplementary Information). Then, tissue sections were placed in hematoxylin solution for 10 min, RT. Subsequently, excess dye was removed by 20 min washing in distilled water. Slides were transferred to the eosin solution for 5 min and washed again in distilled water for 1 min. The following steps of tissue sections dehydration are included in Table S3 in Supplementary Information. Finally, dibutylphthalate polystyrene xylene (DPX) was used as a mounting medium before covering with a coverslip. Images were obtained using an Olympus CX41 microscope equipped with Motic Images Plus 3.0 software. Image analysis was carried out using Adobe Photoshop CC software.

### 4.14. Statistical Analysis

Statistical analysis was carried out using GraphPad Prism ver. 8.0. All results are presented as means ± standard deviation for in vitro research and as means ± standard errors of means for in vivo research. Differences between control and test samples were assessed with one-way ANOVA of variance with Dunnett post-hoc test. A *p*-value of <0.05 was considered statistically significant between groups and is displayed as: *,^-*p* < 0.05; **,^^-*p* < 0.01; ***, ^^^-*p* < 0.001. In the case of the sign (*), it is a comparison between the control sample, not treated with the compound and the test sample incubated with the D2AAK1 in selected concentrations, while the sign (^) means the comparison between the incubation times—48 h and 96 h. All in vitro experiments were carried out in triplicate. ImageJ software was used for image processing, while Adobe Photoshop CC software was used to create figures.

## 5. Conclusions

The significance of results presented here is a consequence of the limited literature data which does not provide extensive experimental evidence about increased hippocampal cell proliferation. Our results confirm that D2AAK1 stimulates neuronal growth and promotes cell survival, which is initiated by the increase of neurotrophic factors, CAMKI kinase activity, and cooperating signals responsible for cell growth and proliferation. Our in vitro results translate into behaviors of mice that have been treated with the compound. in vivo studies confirmed an improvement of memory in mice that may be associated with increased hippocampal neuron proliferation.

In conclusion, D2AAK1, a multi-target ligand of aminergic GPCRs, may provide a new therapeutic option for the treatment of cognitive symptoms of schizophrenia as well as in memory disturbances in neurodegenerative diseases and other CNS disorders.

## Supplementary Materials

Supplementary materials can be found at https://www.mdpi.com/1422-0067/21/22/8849/s1.

## Abbreviations

AD	Alzheimer’s disease
Akt	serine/threonine protein kinase
Bcl-2	B-cell lymphoma 2
BDNF	Brain-Derived Neurotrophic Factor
BrdU	Bromodeoxyuridine
CaMKI	calcium/calmodulin-dependent protein kinase I
CaMKII	calcium/calmodulin-dependent protein kinase II
CDNF	Cerebral Dopamine Neurotrophic Factor
CNS	Central Nervous System
CREB	cAMP Response Element Binding Protein
Erk1/2	extracellular signal-regulated protein kinases 1 and 2
GPCR	G protein-coupled receptor
HT-22	mouse hippocampal neuronal cell line
IGF-IRβ	Insulin-Like Growth Factor-I
MANF	Mesencephalic Astrocyte Derived Neurotrophic Factor
mEPM	modified elevated plus maze test
mTOR	mammalian target of rapamycin kinase
NF-κB	nuclear factor kappa-light-chain-enhancer of activated B cells
NGF	Nerve Growth Factor
NOR	novel object recognition
Trk	tropomyosin receptor kinase
TrkA	tropomyosin receptor kinase A
SH-SY5Y	human neuroblastoma derived cell line
