# Supplementary material for: The Antipsychotic D2AAK1 as a Memory Enhancer for Treatment of Mental and Neurodegenerative Diseases
Source: Int J Mol Sci. 2020 Nov 23;21(22):8849. doi: 10.3390/ijms21228849 (PMC7700684; doi:10.3390/ijms21228849)
Supplement: Supplementary file 1 [file ijms-21-08849-s001.pdf]

Supplementary Information

The antipsychotic D2AAK1 as a memory enhancer for treatment of mental and neurodegenerative diseases

Oliwia Koszła [1]*, Przemysław Sołek [2], Sylwia Woźniak [1], Ewa Kędzierska [3], Tomasz M. Wróbel [1,4], Magda Kondej [1], Aneta Archała [5], Piotr Stępnicki [1], Grażyna Biała [3], Dariusz Matosiuk [1] and Agnieszka A. Kaczor [1,6,*]

[1] Department of Synthesis and Chemical Technology of Pharmaceutical Substances with Computer Modeling Laboratory, Faculty of Pharmacy, Medical University of Lublin, 4A Chodzki St., 20-093 Lublin, Poland, koszlaoliwia@gmail.com; sylwiawozniak@umlub.pl; tomasz.wrobel@umlub.pl; magda.kondej@onet.pl; piotrstepnicki93@gmail.com; dariusz.matosiuk@umlub.pl; agnieszka.kaczor@umlub.pl

[2] Department of Biotechnology, Institute of Biology and Biotechnology, University of Rzeszow, 1 Pigonia St., 35-310 Rzeszow, Poland, pp.solek@gmail.com

[3] Department of Pharmacology and Pharmacodynamics, Faculty of Pharmacy, Medical University of Lublin, 4A Chodzki St., 20-093 Lublin, Poland, ewa.kedzierska@umlub.pl; grazyna.biala@umlub.pl

[4] Department of Drug Design and Pharmacology, University of Copenhagen, Universitetsparken 2, DK-2100 Copenhagen, Denmark

[5] Department of Biopharmacy, Medical University of Lublin, 4A Chodzki St., 20-093 Lublin, Poland, aneta.archala94@gmail.com

[6] School of Pharmacy, University of Eastern Finland, Yliopistonranta 1, P.O. Box 1627, FI-70211 Kuopio, Finland

* Correspondence: koszlaoliwia@gmail.com, agnieszka.kaczor@umlub.pl; Tel.: +48 81 448 7273

Synthesis and spectra of 3-((4-(5-methoxy-1*H*-indol-3-yl)-3,6-dihydropyridin-1(2*H*)-yl)methyl)quinolin-2(1*H*)-one (D2AAK1)

All reagents were purchased from commercial suppliers and used without further purification. NMR spectra were recorded on a Bruker AVANCE III 600 MHz spectrometer equipped with a BBO Z-gradient probe. Spectra were recorded using DMSO-*d6* as a solvent with a non-spinning sample in 5 mm NMR-tubes. Chemical shifts were expressed in parts per million (ppm) referenced to the solvent signal. High resolution mass spectra (HRMS) were acquired on a Bruker microTOF-Q II mass spectrometer with electrospray ionization (ESI). Data were processed using MestReNova v.14.0.0 and Compass Data Analysis software.

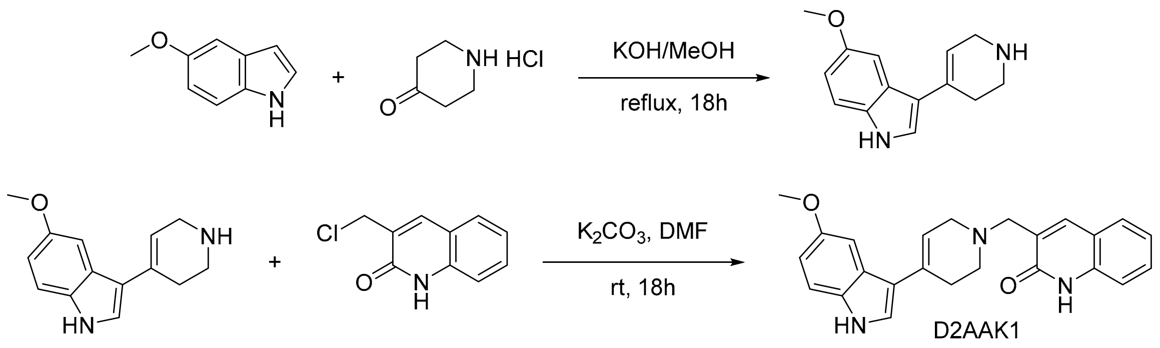

Scheme 1. Synthesis of D2AAK1.

3-((4-(5-methoxy-1*H*-indol-3-yl)-3,6-dihydropyridin-1(2*H*)-yl)methyl)quinolin-2(1*H*)-one

5-methoxyindole (1.47 g, 10 mmol) was dissolved in methanol (40 mL) containing KOH (2.24 g). Piperidin-4-one hydrochloride (1.42 g, 10.50 mmol) was added to the resulting solution and the mixture was heated at reflux for 18 h. The reaction was allowed cool to room temperature and water (50 mL) was added. Precipitated product was filtered and washed in succession with water, methanol and ether to afford 5-methoxy-3-(1,2,3,6-tetrahydropyridin-4-yl)-1*H*-indole as a yellow solid (0.91 g, 40%). Obtained intermediate (0.23 g, 1 mmol) was stirred with 3-(chloromethyl)quinolin-2(1*H*)-one (0.20 g, 1.05 mmol) in DMF (4 mL) with K_2CO_3 (0.14 g, 1 mmol) at room temperature for 18 hours. The reaction mixture was filtered and the solvent was removed in vacuo. Obtained residue was first washed with water and then dissolved in acetone. Insoluble impurities were removed by filtration and the filtrate was stripped of solvent to afford the title compound as a yellow solid (154 mg, 40%).

^{1}H NMR (600 MHz, DMSO) δ 11.82 (s, 1H), 10.97 (d, *J* = 2.7 Hz, 1H), 7.93 (s, 1H), 7.72 (dd, *J* = 7.9, 1.4 Hz, 1H), 7.47 (ddd, *J* = 8.4, 7.2, 1.4 Hz, 1H), 7.36 (d, *J* = 2.6 Hz, 1H), 7.32 (d, *J* = 8.2 Hz, 1H), 7.28 (d, *J* = 8.8 Hz, 1H), 7.26 (d, *J* = 2.4 Hz, 1H), 7.17 (ddd, *J* = 8.1, 7.2, 1.1 Hz, 1H), 6.76 (dd, *J* = 8.8, 2.4 Hz, 1H), 6.13 – 6.09 (m, 1H), 3.77 (s, 3H), 3.53 (s, 2H), 3.27 – 3.22 (m, 2H), 2.73 (t, *J* = 5.7 Hz, 2H), 2.59 – 2.56 (m, 2H). ^{13}C NMR (151 MHz, DMSO) δ 162.4, 154.1, 138.4, 137.1, 132.6, 130.5, 130.2, 130.0, 128.1, 125.4, 123.8, 122.2, 119.8, 117.9, 116.2, 115.2, 112.8, 111.7, 102.7, 56.1, 55.9, 53.5, 50.7, 29.2.

HRMS (ESI) calc. for $C_{24}H_{24}N_3O_2^+$ = 386.1863 found 386.1887.

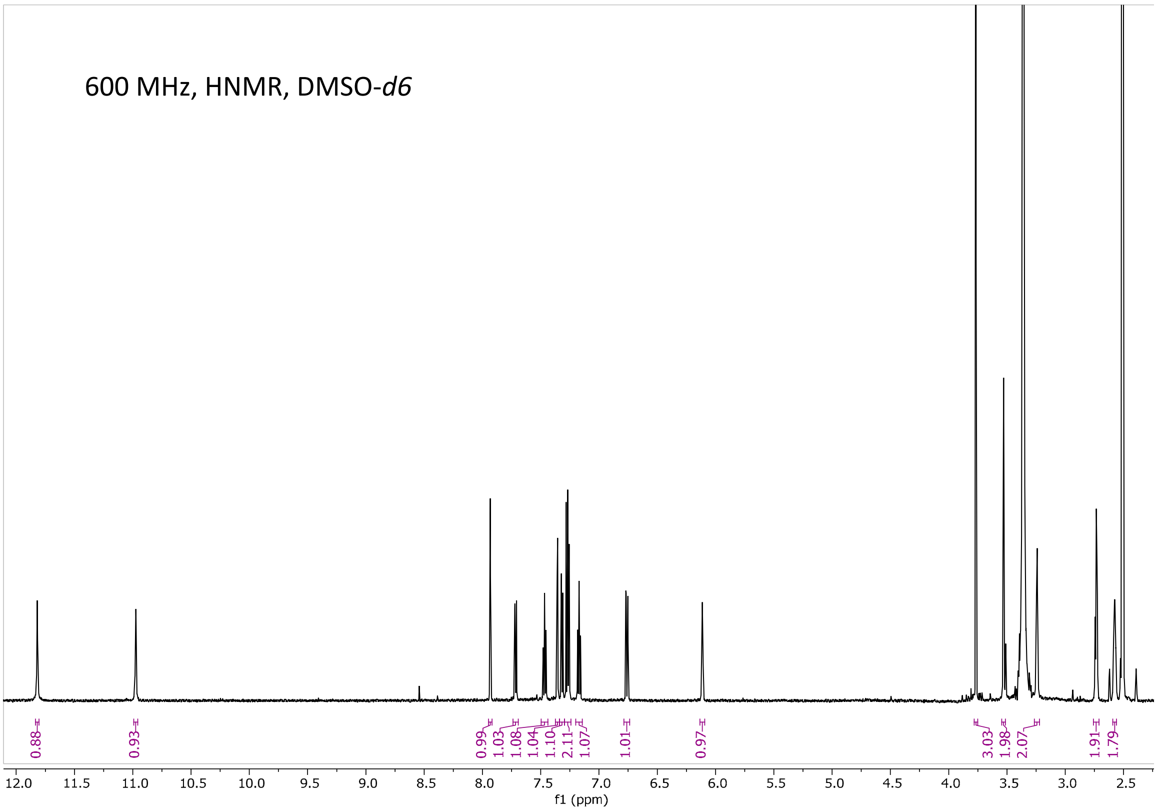
600 MHz, HNMR, DMSO-d6
0.88
0.93
0.99
1.03
1.08
1.04
1.10
2.11
1.07
1.01
0.97
3.03
1.98
2.07
1.91
1.79
12.0 11.5 11.0 10.5 10.0 9.5 9.0 8.5 8.0 7.5 7.0 6.5 6.0 5.5 5.0 4.5 4.0 3.5 3.0 2.5
f1 (ppm)

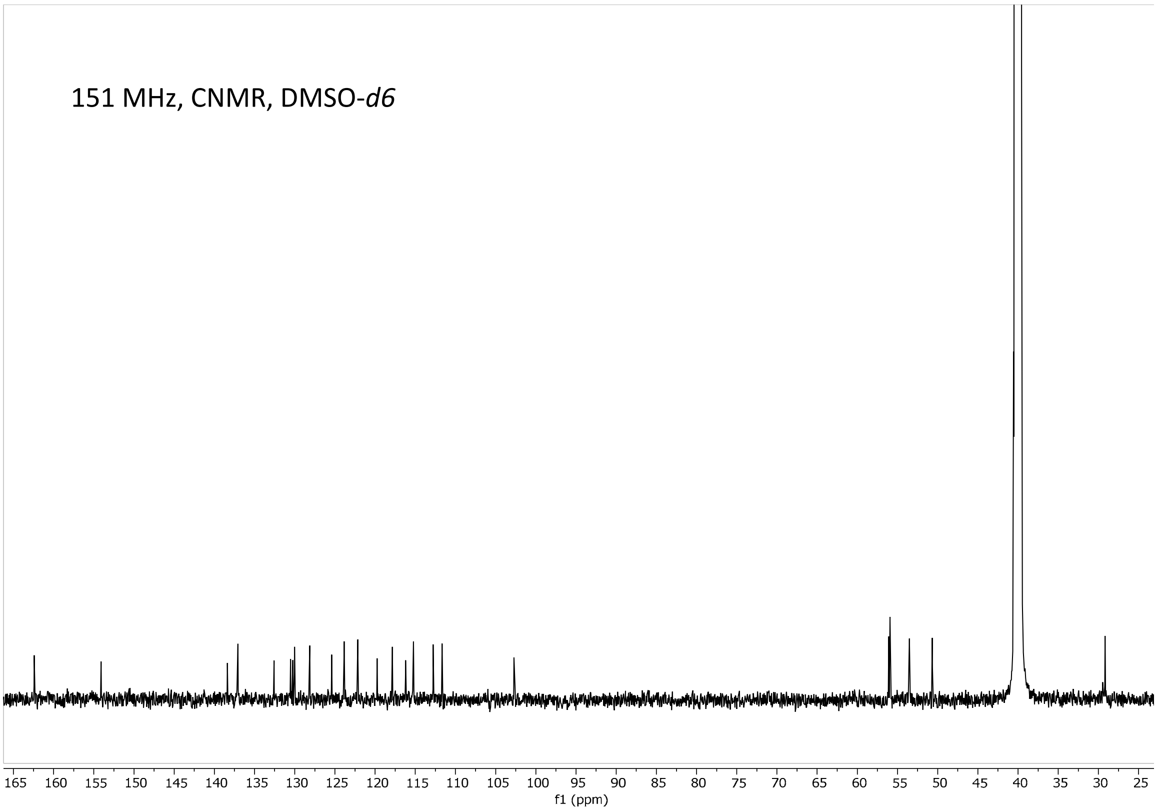
151 MHz, CNMR, DMSO-d6
165 160 155 150 145 140 135 130 125 120 115 110 105 100 95 90 85 80 75 70 65 60 55 50 45 40 35 30 25
f1 (ppm)

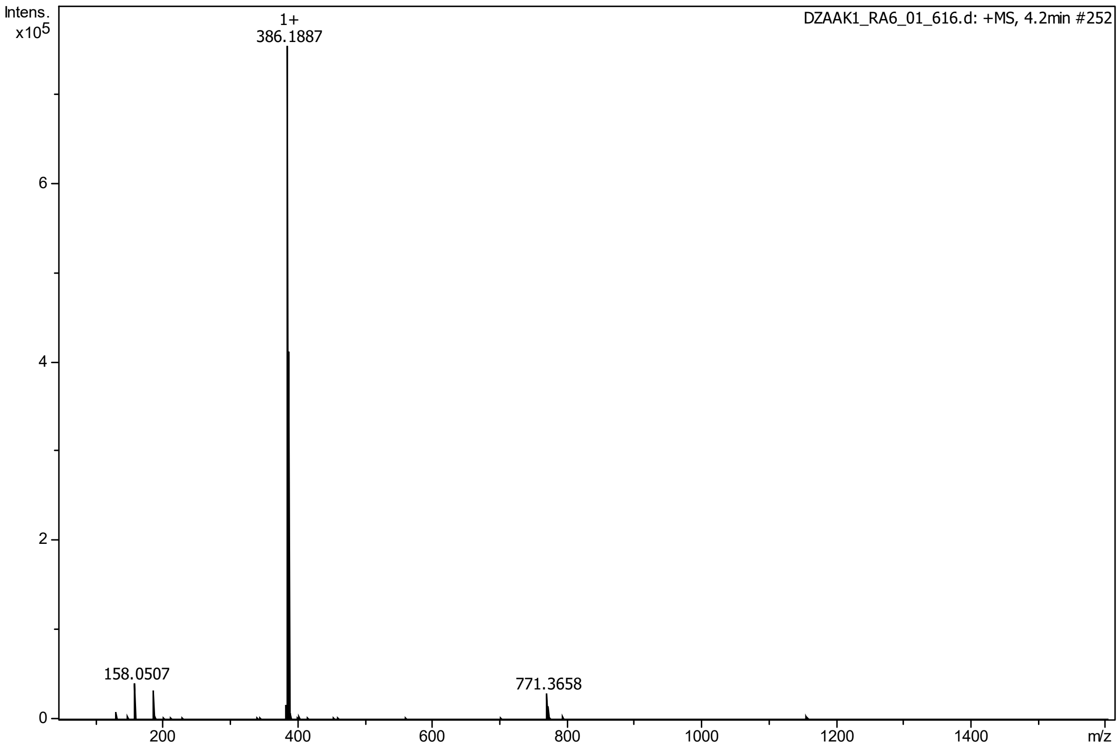

Table S1. Diagram of the procedure for embedding tissue preparations.

Reagent	Incubation time
4% formalin buffer	24h
alcohol 50%	24h
alcohol 70 %	24h
alcohol 96 %	24h
absolute alcohol	1 h
absolute alcohol	1 h
absolute alcohol + xylene	1h
xylene I	20 min
xylene II	20 min
paraplast I	12 h
paraplast II	24h

Table S2. Diagram of the procedure for the tissue sections hydration.

Reagent	Incubation time
xylene I	3 min
xylene II	3 min
absolute alcohol I	3 min
absolute alcohol II	3 min
alcohol 96 % I	3 min
alcohol 96 % II	3 min
alcohol 70 %	3 min
alcohol 50 %	3 min
distilled water	3 min

Table S3. Diagram of the procedure for the tissue sections dehydration.

Reagent	Incubation time
alcohol 70 %	3 min
alcohol 96 % I	3 min
alcohol 96 % II	3 min
absolute alcohol I	3 min
absolute alcohol II	3 min
xylene	3 min
